# Mouse Tumor-Bearing Models as Preclinical Study Platforms for Oral Squamous Cell Carcinoma

**DOI:** 10.3389/fonc.2020.00212

**Published:** 2020-02-25

**Authors:** Qiang Li, Heng Dong, Guangwen Yang, Yuxian Song, Yongbin Mou, Yanhong Ni

**Affiliations:** ^1^Central Laboratory, Nanjing Stomatological Hospital, Medical School of Nanjing University, Nanjing, China; ^2^Department of Oral Implantology, Nanjing Stomatological Hospital, Medical School of Nanjing University, Nanjing, China

**Keywords:** mouse models, OSCC, chemical carcinogen-induced, transplanted, xenograft, syngeneic, HPV, genetically engineered models

## Abstract

Preclinical animal models of oral squamous cell carcinoma (OSCC) have been extensively studied in recent years. Investigating the pathogenesis and potential therapeutic strategies of OSCC is required to further progress in this field, and a suitable research animal model that reflects the intricacies of cancer biology is crucial. Of the animal models established for the study of cancers, mouse tumor-bearing models are among the most popular and widely deployed for their high fertility, low cost, and molecular and physiological similarity to humans, as well as the ease of rearing experimental mice. Currently, the different methods of establishing OSCC mouse models can be divided into three categories: chemical carcinogen-induced, transplanted and genetically engineered mouse models. Each of these methods has unique advantages and limitations, and the appropriate application of these techniques in OSCC research deserves our attention. Therefore, this review comprehensively investigates and summarizes the tumorigenesis mechanisms, characteristics, establishment methods, and current applications of OSCC mouse models in published papers. The objective of this review is to provide foundations and considerations for choosing suitable model establishment methods to study the relevant pathogenesis, early diagnosis, and clinical treatment of OSCC.

## Introduction

Oral squamous cell carcinoma (OSCC) is one of the most common human malignancies and endangers human health, and it accounts for 40% of all head and neck squamous cell carcinoma (HNSCC) cases (1, 2). Although cancer treatments have developed rapidly, the 5-year survival rate for OSCC is still only remained at 50% over the last few decades (3–5). Given the high incidence and poor prognosis of OSCC, an increasing number of researchers are conducting in-depth investigations into the pathogenesis and potential therapeutic targets of OSCC (6–10).

Researches on OSCC can be performed both *in vitro* and *in vivo*. Although experiments *in vitro* have the advantages of relative simplicity, species specificity, convenience, and automation, the extrapolation of *in vitro* results to predict the behavior of tumors in intact organisms is challenging (11). By contrast, experiments *in vivo* using animal models are representative of whole organisms and the use of animal models can avoid issues related to safety, ethics, and extended research cycles that arise in human experiments. Moreover, animal models can accurately reflect the tumor microenvironment—which includes a variety of cytokines, infiltrating immune cells, tumor stroma, and blood vessels—and significant tumor biological behaviors, such as invasion and metastasis. Therefore, a suitable animal model is a prerequisite for clarifying the initiation and progression of OSCC.

Previous researches have revealed that several kinds of animals can be used to establish OSCC models, including hamsters, rats, mice, dogs, and cats (12–17). As early as 1954, squamous cell carcinoma (SCC) of the cheek was induced in hamsters by 9,10-dimethyl-1,2-benzanthracene (DMBA), and hamster SCC exhibits many similarities to human OSCC, including the morphology, histology, infiltration and metastasis, expression of biomarkers, and genetic and epigenetic alterations (18–24). Nevertheless, this model also has its shortcomings, for example, the humans lack cheek pouches, and hamster cheek pouches have inadequate lymphatic drainage (25). Moreover, non-murine models have also been used for the research of OSCC etiology, treatment, and tumor–immune system interactions (17). For example, the metastasis and bone invasion of OSCC has been studied in cats, which can be used to mimic highly malignant OSCC (16, 26, 27), and OSCC has also been researched using dogs for identifying risk factors associated with survival in dogs with non-tonsillar OSCC (15). However, there are fewer reagents available to study dogs and cats, and species-specific drug metabolism and solubility issues are challenging to solve with these models (28).

Rodents other than hamsters, especially rats and mice, are the most commonly used animals in OSCC modeling. The rats are larger but correspondingly more expensive, and there are few immunodeficient or genetically engineered rats. The mouse is characterized by small size, a propensity to breed in captivity, a lifespan of 3 years, extensive physiological and molecular similarities to humans, and an entirely sequenced genome (29). The abundance of particular types of mice, such as immunodeficient mice, genetically engineered mice, and humanized mice, provides a variety of new platforms for the establishment of OSCC models. Therefore, OSCC mouse models have attracted increasing attention from numerous scholars in the OSCC research field.

**Figure 1:**
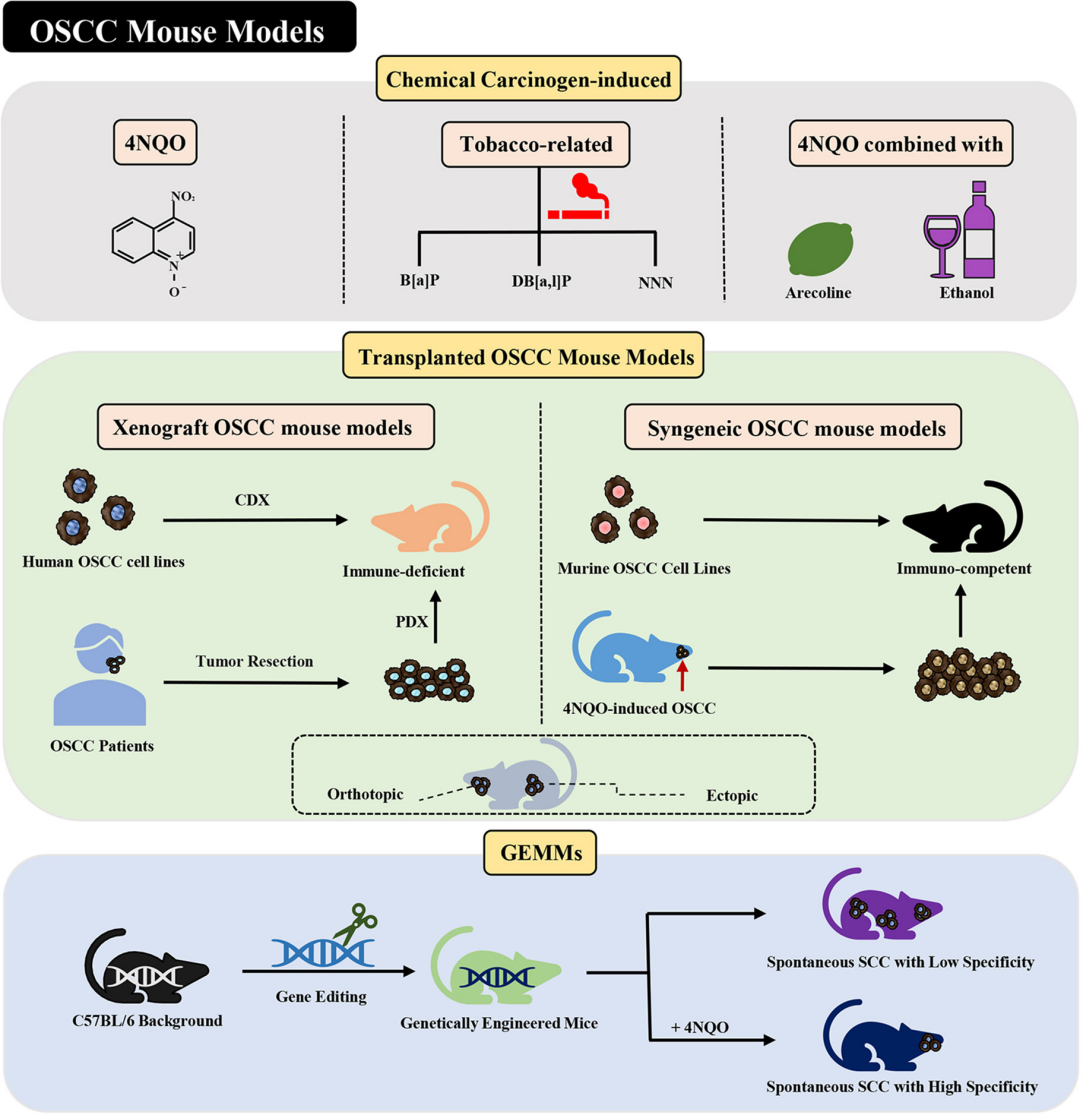
Current mouse models of OSCC. Various methods of establishing OSCC mouse models can be divided into three categories: chemical carcinogen-induced, transplanted, and genetically engineered mouse models. Adhesions: OSCC, oral squamous cell carcinoma; 4NQO, 4-nitroquinoline-1-oxide; B[a]P, Benzo[a]pyrene; DB[a,l]P, Dibenzo[a,l]pyrene; NNN, N'-nitrosonornicotine; CDX, cell-derived xenograft; PDX, patient-derived xenograft; GEMMs, genetically engineered models; SCC, squamous cell carcinoma.

This review focuses on several aspects of OSCC mouse models and is organized by the three main methods by which these models are established: chemical carcinogen-induced, transplanted, and genetically modified OSCC mouse models (Figure 1). Details are provided on the selection and establishment of OSCC mouse models and differences in their mechanisms, characteristics, establishment methods, and applications. This review aims to provide a reference and direction for researchers who are working toward conquering OSCC.

## Chemical Carcinogen-Induced Mouse Models

The main risk factors for OSCC include tobacco, alcohol, long-term chewing of betel quid, human papillomavirus (HPV), and oral lichen planus (especially the erosive form) (30, 31). There are more than 60 carcinogens in cigarette smoke and 16 in unburned tobacco, including tobacco-specific nitrosamines [such as 4-(methylnitrosamino)-1-(3-pyridyl)-1-butanone (NNK) and N'-nitrosonornicotine (NNN)], polycyclic aromatic hydrocarbons (PAHs, such as benzo[a]pyrene), and aromatic amines (such as 4-aminobiphenyl) (32). These chemicals have been reported to induce DNA adducts and correlate with a predisposition to cancer. DNA adducts—the result of covalent binding between DNA and carcinogens, related substances, or their metabolites—are central in the carcinogenesis caused by these agents (32, 33).

Knowing the above-mentioned risk factors for OSCC, researchers have induced murine oral carcinogenesis using a variety of chemical carcinogens, such as 4-nitroquinoline-1-oxide (4NQO) (34), Benzo[a]pyrene (B[a]P) (35), Dibenzo[a,l]pyrene (DB[a,l]P) (36, 37), NNN (37), and combination of 4NQO and arecoline or ethanol (38, 39). The exposure of these chemical carcinogens to the oral cavity of mice can naturally produce a primary OSCC mouse model.

### 4NQO-Induced OSCC Mouse Model

#### Mechanism of 4NQO-Induced OSCC Mouse Model

**Figure 2:**
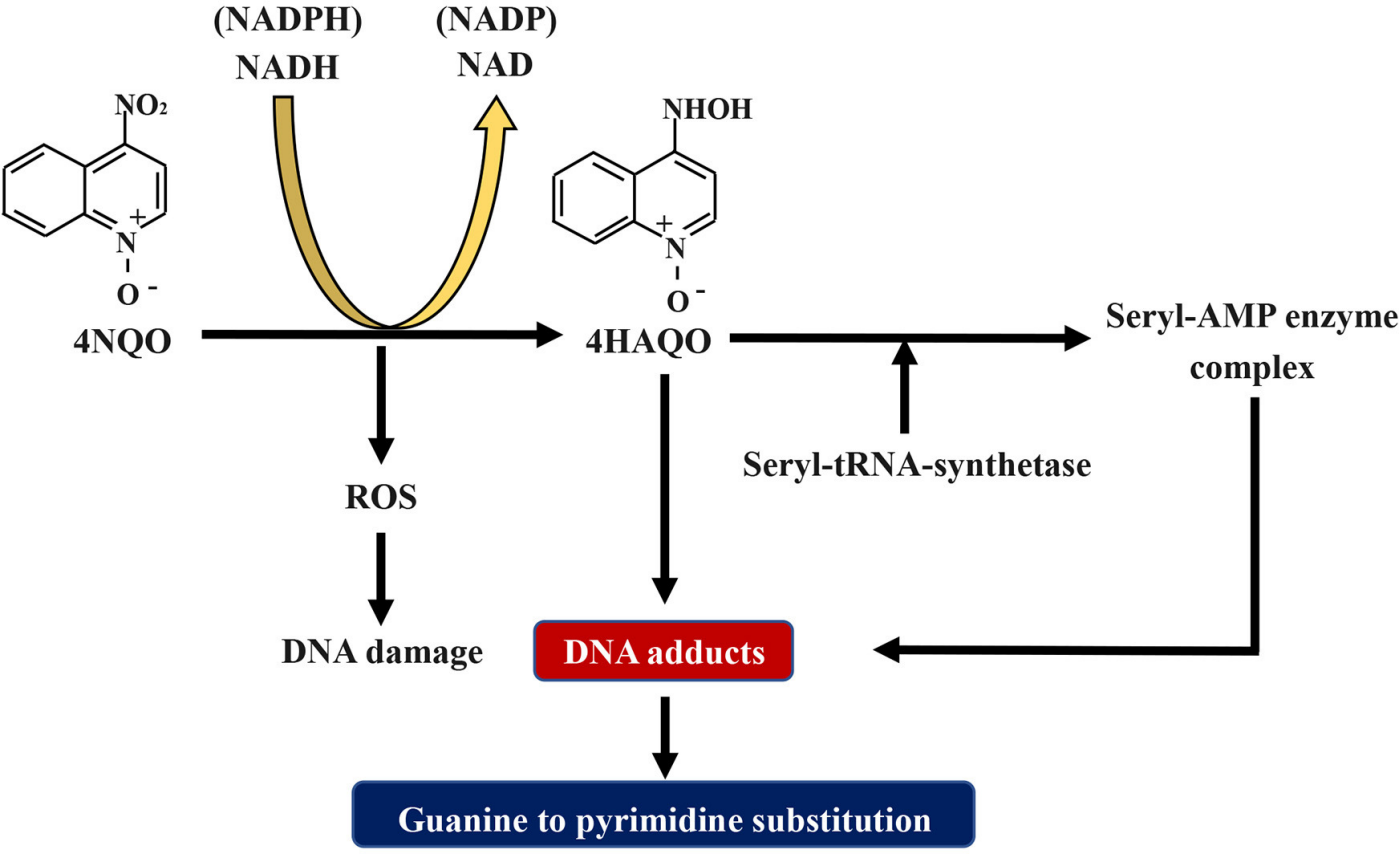
Schematic diagram about the mechanism of 4NQO carcinogenesis. 4NQO is firstly reduced to 4HAQO by NADH and NAD(P)H, while 4HAQO can be acetylated by seryl-tRNA synthetase to form a seryl-AMP enzyme complex. The resultant 4HAQO and seryl-AMP enzyme complex are carcinogenic metabolites that induce the formation of DNA adducts. Mutations caused by these DNA adducts lead to guanine-to-pyrimidine substitution. Adhesions: 4NQO, 4-nitroquinoline-1-oxide; 4HAQO, 4-hydroxy amino quinoline-1-oxide.

The compound 4NQO is an aromatic amine heterocyclic compound and a precursor carcinogen that is typically manufactured for research purposes. Currently, it is the most recognized and frequently used chemical carcinogen for establishing chemical carcinogen-induced OSCC mouse models (40). Researches have proved that 4NQO mimics tobacco and plays a carcinogenic role by causing intracellular oxidative stress, DNA adduction, mutagenesis, and tumor induction (Figure 2) (41–43). It has been revealed as a potent inducer of intracellular oxidative stress by generating reactive oxygen species (ROS) (44, 45), which is related to carcinogenesis that is caused by DNA damage (46–49). Although 4NQO is not a direct carcinogen, it is an electrophilic species produced by metabolism in the body and undergoes an irreversible reaction with the nucleophilic part of DNA, which ultimately introduce mutations. The carcinogenic action of 4NQO is initiated by the enzymatic reduction of its nitro group: first, 4NQO is reduced to 4-hydroxy amino quinoline-1-oxide (4HAQO) by NADH and NAD(P)H, which thus act as 4NQO nitroreductase and quinone reductase, respectively (50). Then, 4HAQO can be acetylated by seryl-tRNA synthetase to form a seryl-AMP enzyme complex (51). The resultant 4HAQO and seryl-AMP enzyme complex are carcinogenic metabolites that induce the formation of DNA adducts. The cellular detoxification of 4NQO is carried out by multidrug resistance protein (MRP) and glutathione S-transferase P (GSTP1-1), which may play essential roles in preventing the initiation and progression events of carcinogenesis (52). The imbalance between the two pathways is the leading cause of tumor induction by 4NQO. Nuclear magnetic resonance (NMR) studies and *in vivo* experiments have revealed that 4HAQO binds preferentially to G residues of DNA (53, 54). Moreover, the third and fourth position of the acetylated metabolite of 4NQO can react with the N2 and C8 positions of guanine (55). Mutations caused by these DNA adducts result in guanine to pyrimidine substitution (56, 57).

#### Characteristics of the 4NQO-Induced OSCC Mouse Model

**Figure 3:**
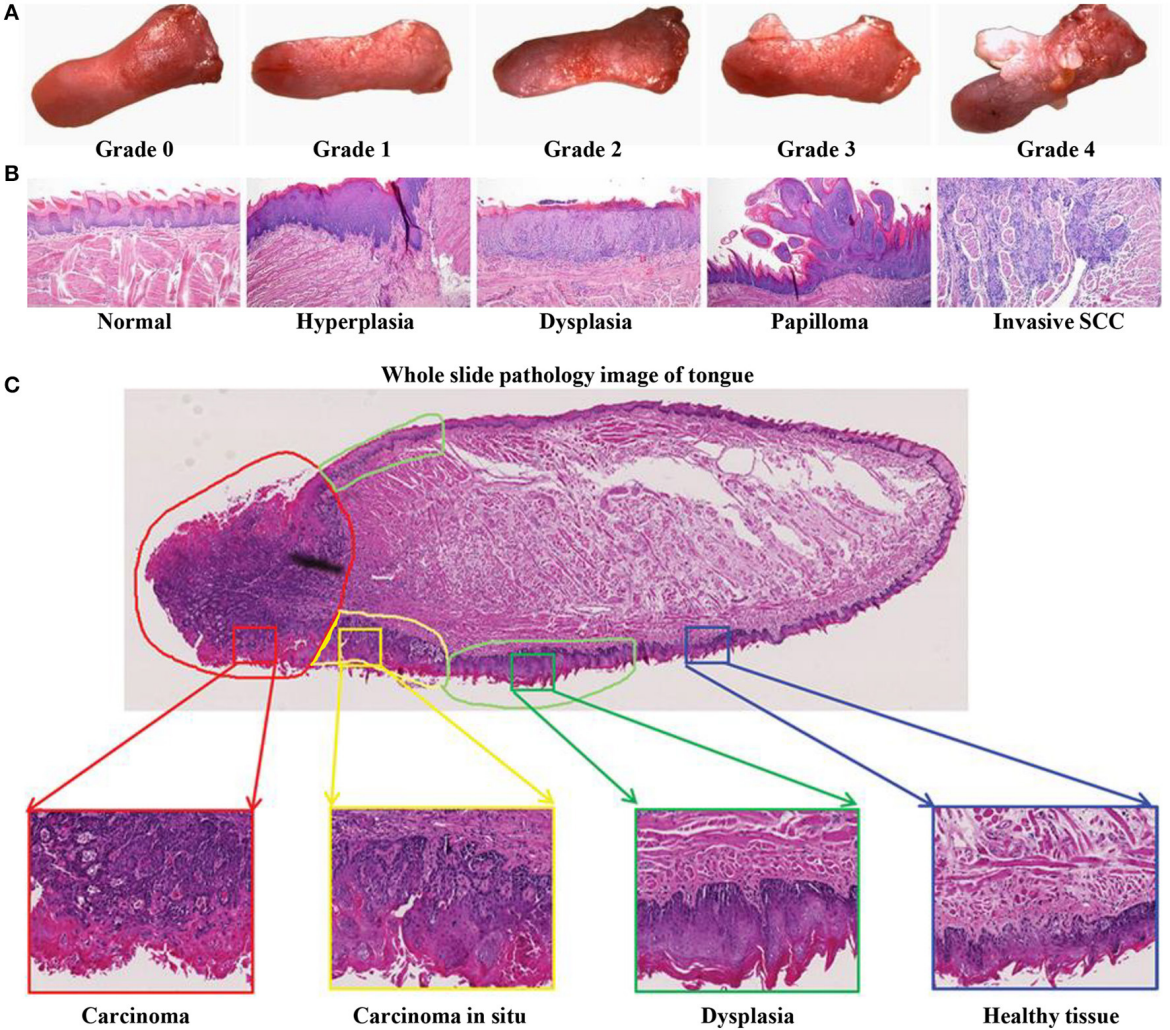
4NQO-induced OSCC mouse model. **(A–C)** Representative gross morphology and pathological stages of the tongue from 4NQO-induced mice. **(A)** The gross tongue lesion grading system (8×), and the severity of the lesions gradually increased from 0 to 4. **(B)** Representative pathological stages of tongue lesions. **(A,B)** Reproduced and modified with permission (59). *Copyright* © *2014, National Academy of Sciences*. **(C)** A whole slice image of tongue specimen contains tongue lesions of dysplasia, carcinoma *in situ*, and carcinoma. Reproduced and modified with permission (60). *Copyright* © *2017, Wiley Online Library*.

The 4NQO-induced mouse model reflects the multistage dynamic carcinogenicity of human OSCC, from dysplasia to invasion. Researchers using this model reported that the gross morphology of lesions changed from mild to severe dysplasia (Figure 3A), and the pathological stages of tongue lesions underwent hyperplasia, dysplasia (mild, moderate, and severe), *in situ* carcinoma, and invasive squamous cell carcinoma (SCC) (Figure 3B) (58). One of the characteristics of this model is that not all mice develop the same lesions at the same time, and a variety of lesions can be seen on the tongue of a single mouse (Figure 3C). In a study using the 4NQO-induced oral cancer pain model, adult female C57BL/6 mice were given 4NQO (100 μg/mL) dissolved in propylene glycol for 16 weeks. The treatment was then switched to regular drinking water for 16–28 weeks. By 28 weeks, pathological changes in the tongue were observed in all mice, of which 20.8% had moderate atypical hyperplasia, 45.9% developed severe atypical hyperplasia and carcinoma *in situ*, and 33.3% progressed to squamous cell carcinoma (61). But the OSCC mouse model established by this method had a low degree of malignancy and rarely underwent metastasis and bone invasion (40).

The molecular events showing in the 4NQO-induced mouse model closely resemble those observed in human HNSCC patients (62, 63). Moreover, the 4NQO-induced OSCC mouse model has been proved effective because it is very similar to the occurrence of human OSCC at the genetic and molecular level. A review of molecular alterations at various stages of 4NQO-induced oral carcinogenesis had been accomplished by Kanojia and Vaidya, which covered apoptosis-related proteins, cell cycle-related proteins, proteins of cell-cell interactions, and cytoskeletal proteins (40). To identify the genetic alterations of 4NQO-induced C57BL/6J mice during the development of lingual SCC, Liu et al. (64) induced C57BL/6J mice with 4NQO (50 mg/L in drinking water) and then harvested lingual mucosa samples from different stages, namely normal tissue (0 week) and early-stage (12 week) and advanced-stage (28 week), respectively. Through microarray and methylated DNA immunoprecipitation sequencing, the result of bioinformatics analysis revealed significant alterations in 63 hub genes and promoter methylation, including Tbp, Smad1, Smad4, Pdpk1, Camk2, Atxn3, and Cdh2. Of the 63 human orthologous genes, 100% were reported to be associated with human cancer, and up to 55.5% were relevant to human oral cancer. Schoop et al. (65) revealed that the immunopathology of the murine oral tumor was similar to that of the described human oral tumor through immunohistochemistry labeled with cyclin D1 and E-cadherin.

The carcinogenic effect of 4NQO starts with its reduction by 4NQO reductase, so the distribution and quantity of 4NQO reductase in different tissues affect the tissue specificity of 4NQO carcinogenesis (66). 4NQO can induce carcinogenesis in many parts of the oral cavity, such as the dorsal tongue, ventral tongue, and palate (67, 68). Additionally, 4NQO passes from the oral cavity through the entire digestive tract, and the esophagus also contains a large amount of 4NQO reductase; therefore, this model can also form esophageal cancer (67, 69).

#### Methods for 4NQO-Induced OSCC Mouse Model

The strains of mice used for the 4NQO-induced OSCC mouse model include immunocompetent C57BL/6, BALB/c, CF-1, and CBA mice (Table 1). Most studies that have applied this method used adult mice aged 6–8 weeks, but Vincent-Chong et al. (76) observed that 92% of old mice (aged 65–70 weeks) developed severe dysplasia/invasive squamous cell carcinoma, while the incidence in young mice (aged 7–12 weeks) was 69%. CBA mice have been reported to be more susceptible to 4NQO induction than C57BL/6 mice (67, 77). Tang et al. (67) used 4NQO in drinking water to induce C57BL/6 and CBA mice and found that both strains developed a variety of precancerous lesions and carcinogenesis in the tongue and esophagus. CBA mice were treated with 50 and 100 μg/mL 4NQO for 8 weeks, and 100% of them developed tongue lesions, and 100 μg/mL 4NQO treatment for 8 or 16 weeks resulted in carcinogenesis of the tongue and esophagus. After 16 weeks of treatment with 100 μg/mL 4NQO, tongue lesions were observed in 100% of C57BL/6 mice, but visible gross lesions were not seen until the following 4–12 weeks.

**Table 1:** Methods of 4NQO-induced mouse models in drinking water for OSCC.

**The concentration of 4NQO (μg /mL)**	**Treatment period (/weeks)**	**Development period (/weeks)**	**Mouse strains**	**References**
50	20	4	BALB/c	(70)
50	16	4 or 8	C57BL/6	(71)
100	16	4, 8, or 10	C57BL/6NCr	(13)
100	16	12	C57BL/6	(61)
100	16	12 or 13	CF-1	(72)
100	8	4, 8, 12, or 16	CBA	(73)
100	8	10	C57BL/6	(74)
100	16	6 or 12	C57BL/6	(75)

Administration of 4NQO can be carried out by topical application or the addition to drinking water, and both methods have successfully established the OSCC mouse model (65, 76, 78–81). Initially, researchers used localized smearing of 4NQO to induce OSCC, which was the same approach used to induce SCC in hamster pouches by DMBA (65, 80). Schoop et al. (65) smeared the tongues of male CBA mice with 4NQO (5 mg/mL) dissolved in propylene glycol for 16 weeks, with three treatments every week. From 24 to 40 weeks, the researchers detected the continuous development of hyperplasia; mild, moderate, and severe dysplasia; and squamous cell carcinoma. The smear method ensures that 4NQO acts on the oral cavity to a great extent and reduces the 4NQO burden in the digestive tract as much as possible. However, some researchers have recently used drinking water containing 4NQO to establish the OSCC model since it is a more natural means of administration, faster to model, and less painful to the mice than the topical application method (76, 78, 79, 81).

As shown in Table 1, the dosage and administration time of 4NQO used by the drinking water method in different studies are diverse. The most commonly used 4NQO concentrations in drinking water to induce OSCC are 50 and 100 μg/mL (61, 70). In general, a higher concentration means the establishment of OSCC is faster. Typically, the mice are exposed to 4NQO in the drinking water for about 16 weeks (treatment period) and then provided with normal drinking water for an additional 6–16 weeks (development period) (76, 78, 79, 81). There is no unified standard for the establishment of 4NQO-induced OSCC mouse models at present. Researchers can adjust these factors according to the purpose of the study. Tang et al. (67) administered different concentrations of 4NQO in drinking water to CBA mice: 100 μg/mL 4NQO for 16 weeks, 100 μg/mL for 8 weeks, 50 μg/mL for 8 weeks, and 20 μg/mL for 8 weeks. Of the four groups, 100% of the first three groups developed oral cavity lesions; the values of the bromodeoxyuridine (BrdUrd) labeling index, which can be used to reflect the degree of malignancy of oral cavity lesions, were 21.0 ± 0.9%, 15.1 ± 0.8%, 10.9 ± 0.8%, and 11.1 ± 0.5%, respectively, for the four groups.

#### Usage of 4NQO-Induced OSCC Mouse Model

Since the similarities of the 4NQO-induced OSCC mouse model in pathogenesis, pathological changes, and host immune activity, and molecular level to those of humans, this model is widely used to study OSCC, especially in developing the biomarkers for early diagnosis and the transformation of the epithelium. Because 4NQO simulates tobacco-related gene mutation and can induce primary OSCC, this model has been used to explore the pathogenesis of OSCC (82). A spontaneous tumor has a more natural tumor microenvironment (76, 83) and can also be used in the study of cytokines (61), mesenchymal stem cells (84), natural killer (NK) cells (85, 86), microbiomes (7), and angiogenesis (87). The murine lesions induced by 4NQO constitute a dynamic and continuous process and can be used to study the early detection and prevention of precancerous OSCC lesions (88–90). The immune activity of this model renders it suitable for researching the changes in tumor immunology in the process and development of tumorigeneses, as well as related subjects such as immunosuppression (91–94) and immunotherapy (34, 95, 96). In conclusion, the 4NQO-induced OSCC mouse model appears to be the best available model for research the diagnostic and prognostic markers for OSCC. However, this model has limited application for studies on the invasion and metastasis of malignant tumors because of its low probability of metastasis and bone invasion.

### Other Chemical Carcinogen-Induced OSCC Mouse Models

#### Tobacco-Related Chemical Carcinogens-Induced OSCC

##### Benzo[a]pyrene (B[a]P)

B[a]P, a member of the PAHs family and a procarcinogen, is widely distributed in tobacco smoke, charcoal-grilled foods, contaminated water, engine exhaust, and soil (97). In a 2-year bioassay of female B6C3F1 mice, Culp et al. (35) compared the effect of coal tar and B[a]P on tumor induction and discovered that the mice fed with 100 ppm B[a]P developed tongue lesions (papillomas and carcinomas) with an incidence of 23/48. However, the main target of B[a]P is the forestomach. Although high doses of B[a]P can induce tongue cancer, the toxicity of B[a]P is high, and the low carcinogenic specificity of B[a]P will produce lesions in other parts of the body, including the liver, lung, forestomach, esophagus, and larynx, which limit its application.

##### Dibenzo[a,l]pyrene (DB[a,l]P)

DB[a,l]P is a potent carcinogen produced in cigarette smoke and has been used to induce carcinogenesis of the lung, skin, mammary gland, and oral cavity in mice and rats (36, 98–100). Guttenplan et al. (36) revealed that a 24 nmol dose of DB[a,l]P by topical application to the oral cavity resulted in neoplasia in 31% of B6C3F1 mice, accompanied by the elevated expression of p53 and COX-2 protein. Furthermore, DB[a,l]P induced ovarian tumors.

##### DB[a,l]P and N'-nitrosonornicotine (NNN)

Guttenplan et al. (37) compared the carcinogenesis and mutations resulting from the administration of DB[a,l]P, NNN, and both NNN and DB[a,l]P. DB[a,l]P (0.16 μmol) was topically applied on the tongue of *lacI* mice three times per week, while NNN (8.46 μmol) was applied two times per week. After induction for 5 weeks and development for 4 weeks, the mice were euthanized. This study revealed that DB[a,l]P + NNN caused the highest percentage of genetic mutations.

#### 4NQO Combined With Other Chemical Carcinogens

##### 4NQO and arecoline

Arecoline is an alkaloid extracted from betel nut and plays an essential role in the progression of oral cancer (101, 102). Chang et al. (38) treated C57BL/6JNarl mice with arecoline, 4NQO, or both arecoline and 4NQO for 8 weeks to induce oral carcinogenesis. They found that 100% of the mice induced by both 4NQO (200 μg/mL) and arecoline (500 μg/mL) developed tongue tumors, while 57 and 0% of the mice exposed to only 4NQO or arecoline developed tongue tumors, respectively. Similar to the results in human studies (103), immunohistochemical analysis revealed the expression of αB-crystallin and Hsp27 in this mouse tumor model. In this model, the mouse oral cavity was exposed to 4NQO and arecoline at the same time, simulating the etiology for oral cancer associated with betel nut.

##### 4NQO and ethanol

Alcohol is another risk factor for oral cancer. Guo et al. (39) treated male wild-type C57BL/6J mice and 5-Lox knockout mice (*Alox5*^*tm*1*Fun*/*J*^) with 100 μg/mL 4NQO in drinking water for 8 weeks, and then in the following 16 weeks, 8% ethanol was provided *ad libitum* as the sole drink water. The incidence of OSCC increased from 20 (no ethanol) to 43% (8% ethanol) in wild-type mice, while fewer cancers were induced in the 5-Lox knockout mice. This study revealed that ethanol could promote 4NQO-induced oral carcinogenesis in mice by activating the 5-LOX pathway of arachidonic acid metabolism.

## Transplanted OSCC Mouse Models

Transplanted mouse models can be more accurately named “animal cultures” because tumor cells or tumor tissues are transferred to mice for culture. As early as 1969, Rygaard (104) successfully transplanted human malignant tumors into nude mice for the first time, which offered a new prospect in the research and application of transplanted mouse models.

### Xenograft OSCC Mouse Models

#### Classifications of Xenograft OSCC Mouse Models

In tumor xenograft mice, tumor tissue or cell lines from one species are propagated in ectopic or orthotopic sites in immunodeficient mice (29). As shown in Table 2, depending on the source of the graft, xenograft mouse models can be divided into cell-derived xenografts (CDX) and patient-derived xenografts (PDX), which are derived from tumor cells cultured *in vitro* and fresh tumor tissues from patients, respectively. Xenograft mouse models can also be divided into ectopic or orthotopic mouse models depending on the tumor location. In ectopic mouse models, tumor cells are subcutaneously injected into the flank or back, while in orthotopic mice, tumor cells are typically transplanted to the tongue of mice.

**Table 2:** Classifications of Xenograft mouse models.

**Basis of classification**	**Classification**	**Advantages**	**Disadvantages**
The source of the graft	CDX	Readily available cell lines, vast published literature for reference, and low cost	Tumor heterogeneity was significantly different from the original tumor tissue
	PDX	Tumor heterogeneity was stably retained	Complicated operation unrepeatable modeling, long construction time, high cost, and unstable success rate
The tumor location	Ectopic	Time-saving and less labor	Tumors formed in ectopic sites Rare metastasis and bone invasion are
	Orthotopic	Develop tumors in the oral region Develop bone invasive and distant metastasis	Harder implantation than ectopic xenograft More painful for mice

#### Characteristics of Xenograft OSCC Mouse Models

A xenograft can be continuously transplanted in the same species or the same strain of animal, and the characteristics of easy replication, a high modeling success rate, and high stability facilitates the generation of abundant experimental mice.

**Figure 4:**
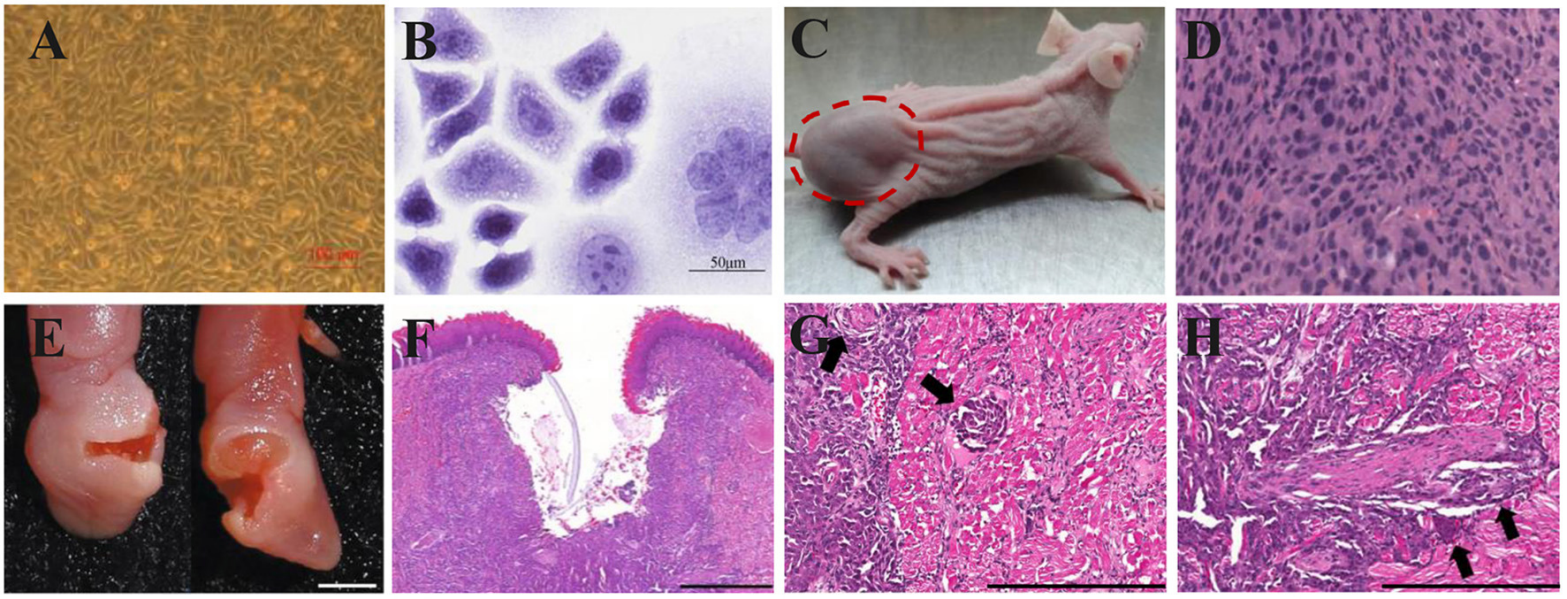
Xenograft OSCC mouse models. **(A–D)** An ectopic mouse model of OSCC. **(A)** OSCC-BD cells overlapped and lost contact inhibition. **(B)** HE staining showed that OSCC-BD cells had malignant characters. **(C)** The nude mice were subcutaneously injected with OSCC-BD cells. Tumor formation was observed, and neoplasms were about 1.0± 0.2 cm in size. **(D)** HE staining showed the neoplasm was typical squamous cell carcinoma. Scale bars, 100 μm **(A)** and 50 μm **(B)**. Reproduced and modified with permission (110). *Copyright* © *2015, Springer Nature*. **(E–H)** An orthotopic mouse model injected into the lateral border of the tongue of non-obese diabetes–severe combined immune deficiency (NOD-SCID) mice via HSC-3 cells. The HSC-3 bearing tumors have **(E,F)** ulcers, **(G)** neural, and **(H)** vascular invasion (arrows). Scale bars, 0.2 cm **(E)**, 400 μm **(F)**, and 200 μm **(G,H)**. Reproduced and modified with permission (111). *Copyright* © *2018, Springer Nature*.

The prominent characteristic of CDX models is high tumor consistency: after inoculating a certain number of tumor cells into mice, the volume and growth rate of the tumor are consistent, the inter-individual difference is low, and the effects on the hosts are identical (Figures 4A–D). But the transplanted tumor microenvironment lacks surrounding tissues, especially the stromal cells, vascular and lymphatic circulation, and immune cells of human origin (Figure 4D) (105, 106). Because different immunodeficient mice are used, the xenograft tissue lacks the structure of a typical immune system, although the immune cells are not absent. HE staining of HSC2-bearing tongues, dissected from athymic nude mice injected with HSC2 cells, revealed an immune reaction that included neutrophils, lymphocytes, and macrophages (107). CDX models can be considered PDX models with too many generations to be traceable. After multiple generations, the heterogeneity in the genetic, histological, and phenotypic characteristics of the tumors causes gradual differences from the original tumor tissue (108). For this reason, the US National Cancer Institute (NCI) retired the NCI-60 panel−60 human tumor cell lines established by NCI—from the drug-screening program (109). PDX models can more accurately reflect the mechanism of tumor occurrence and development in patients. In contrast to the traditional CDX models, PDX models preserve the genotypic and phenotypic diversity of tumor tissue to reflect the characteristics of the original tumor genuinely. PDX models also maintain tumor stromal cells and tumor microenvironment (Figures 4F–H). In a previous study, HE and immunohistochemistry (IHC) staining revealed that the histopathology and IHC were highly consistent between the PDX models and the corresponding patients (112). However, after the secondary transfer of the PDX tumor, the tumor stroma disappeared rapidly and was replaced by mouse interstitial cells (113).

The ectopic OSCC model can be established by subcutaneous injection with little effort; the operation is simple, and the observation of ectopic tumors and the measurement of tumor volume are also intuitive. Furthermore, the growth of subcutaneous tumors is not limited by the oral cavity size of mice (Figure 4C). However, the anatomical structures of the oral cavity and the subcutaneous tissue have some differences, such as the vascular distribution, lymphatic reflux, and bone adjacency. The orthotopic model can provide an experimental environment more similar to the original tumor environment than the ectopic model (28). Besides, more aggressive behaviors of regional and distant metastasis were discovered in the orthotopic models (114, 115). However, the growth of intra-oral tumors in the orthotopic model will not only cause pain but also hinder the diet of mice, which is against humanitarianism. Fitch et al. (116) firstly established an orthotopic model of human OSCC cell lines. Tumor cells were extracted from tumors growing subcutaneously and then injected into the tongue of nude mice. Equal tumorigenicity of tumor cells was finally detected in oral and subcutaneous tissues. In contrast, Myers et al. (117) compared the tumorigenicity of three human OSCC cell lines, Tu159, Tu167, and MDA1986, in the orthotopic tongue and ectopic subcutaneous tissues. It was found that all three types of SCC were more likely to develop in orthotopic than ectopic. The local tumor growth, regional lymph node metastases, and distant visceral metastases of the established orthotopic nude mouse model showed similar histopathology and biological characteristics to the patient's primary tumor.

#### Methods of Xenograft OSCC Mouse Models

The mice used in this model need to be immunodeficient because xenografts can otherwise trigger an immune response and cause a rejection reaction and graft-vs.-host disease (GvHD). The commonly used immunodeficient mice are athymic nude mice, severe combined immune deficiency (SCID) mice, non-obese diabetes–severe combined immune deficiency (NOD-SCID) mice, and NOD-*Prkdc*^*scid*^*IL2rg*^*null*^ mice, such as NOD.Cg-*Prkdc*^*scid*^*Il2rg*^*tm*1*Sug*^/ShiJic mouse (NOG) and NOD.Cg-*Prkdc*^*scid*^*Il2rg*^*tm*1*Wjl*^/SzJ mouse (NSG). Different gene mutations cause varying degrees of immunodeficiency in the four kinds of mice. Comparisons by various researchers have revealed that the mice with the highest potential for successfully engrafting and investigating human cancers are the immunodeficient *IL2rg*^null^ mice, followed by NOD-SCID mice, SCID mice, and athymic nude mice, in descending order of efficacy (Table 3) (118–120).

**Table 3:** Four kinds of immune-deficient mice (118–125).

**Mouse species**	**Genetic backgrounds**	**Immunodeficiency**	**Life span**	**Main features**
Athymic nude mouse	Homozygous nu/nu mutant mice; spontaneous deletion in the Foxn1 gene	Lack of functional T lymphocytes	18 months to 2 years	Hairless, naked, and athymic
SCID mouse	A spontaneous mutation in C.B-I7Icr (C.B17) mice termed “SCID” (Prkdc^scid^), or express the Rag1^null^ or Rag2^null^ mutations	Lack of mature T and B lymphocytes	Above 1 year	“leaky,” can generate a certain degree of functional T and B cells with the increase of age
NOD-SCID mouse	Backcross the SCID mutation onto NOD mouse strain background	Lack of functional T and B lymphocytes. Lower NK cell activity, reduce levels of macrophage activation, abnormal DCs development and an absence of hemolytic complement	8 to 9 months	Less “leaky”; has spontaneous lymphoma
NOD-*Prkdc^*scid*^IL2rg^*null*^* mouse	NOD genetic background, Prkdc^scid^, and a targeted mutation in the IL2-receptor common gamma chain gene (IL2rg^null^)	Lack T, B, and NK cells and have functionally impaired DCs and macrophages	1.5 years	No B lymphocyte leakage

In ectopic mouse models, tumor cells are subcutaneously injected into the flank or back, while in orthotopic mice, tumor cells are mainly transplanted into the tongue of mice. Moreover, the orthotopic OSCC mouse model can also be established at the floor-of-mouth (FOM) region (126–128). Transplanting tumor cells into the FOM region allowed the tumor to occur in the submental region of the jaw rather than in the mouth, in order to create a tumor that was easier to surgically remove and avoided inevitable complications if a trans-oral resection was attempted (127).

CDX models are developed using established human-derived cell lines, such as the human oral carcinoma cell lines SCC9 (1 × 10^7^ cells) (129), UM-SCC47 (1 × 10^6^ cells) (130), CAL27-DsRed (0.5 × 10^6^ cells in 40 μL per tongue) (131), and UMSCC2-DsRed (0.5 × 10^6^ cells in 40 μL per tongue) (131) (Table 4). The construction of PDX models has four phases (P): surgical removal of the tumor (P_0_), engraftment (P_1_), expansion (P_2_), and treatment (P_3_…P_n_). The tumor tissue obtained from surgery is cut into fragments (diameter of about 1 mm), mixed with Matrigel, and then transplanted into immunodeficient mice. When the tumor grows to a specific size, the tumor is removed and then re-transplanted for tissue expansion. The growth rate of each cell line or tumor tissue derived from different patients varies, and the time required for the successful construction of the transplanted tumor has differed among studies. Therefore, the endpoint of tumor growth is evaluated by the volume of the tumor (Table 4).

**Table 4:** Xenograft OSCC mouse models.

**Classification**	**Cell lines / tissue source**	**End point**	**Mouse strains**	**Ref**.
PDX	Patients with HNSCC	Xenografts reached 100 mm^3^ to 200 mm^3^	NSG mice	(130)
CDX	UM-SCC47 cells (1 × 10^6^ cells)	Xenografts reached 100 mm^3^ to 200 mm^3^	Nude-Foxn1^null^ mice	(130)
CDX;Orthotopic	CAL27-DsRed cells (0.5 × 10^6^ cells in 40 μl per tongue) UMSCC2-DsRed cells (0.5 × 10^6^ cells in 40 μl per tongue)	CAL27 tumors grew up to 25 ± 6 mm^3^ by day 31 UMSCC2 tumors grew up to 20 ± 5 mm^3^ by day 31	Nude mice (NCr ^nu/nu^)	(131)
PDX; Ectopic (bilaterally subcutaneous)	Three patients with HNSCC	A volume of 2,000 mm^3^	SCID mice	(132)
PDX; Ectopic (in the flank)	HNSCC patients (25 mg of tumor/mouse)	A volume of 1 cm maximum diameter	NOD/SCID gamma mice	(133)
PDX; Ectopic (subcutaneous)	OSCC of right retromolar trigone, T_3_N_0_M_0_ and tongue—SCC, T_1_N_2b_	About 30 days	SCID mice	(134, 135)
PDX; Ectopic (subcutaneously on the flank)	Two patients with oral cancer T_4_N_0_M_0_ and T_2_N_2_M_0_	A volume of 1,000–1,500 mm^3^	Nude mice	(136)

#### Usage of Xenograft OSCC Mouse Models

The development of xenograft mouse models has enabled the study of human tumor tissues and cell lines *in vivo* in immunodeficient mice. In light of the advantages as mentioned above of “animal cultures” of tumor cells or tissues *in vivo*, most antineoplastic drugs have been tested using xenograft mouse models in the preclinical stage. Compared with CDX models, PDX models are more advanced preclinical oncology models for new drug development and can achieve better preclinical drug efficacy testing and analysis. As the tumor model closest to the actual conditions of the human body, the PDX model can provide personalized drug guidance for a single patient and realize precision therapy for a tumor. In the field of OSCC research, xenograft OSCC mouse models are used for screening drugs (137–139) and studying drug resistance, such as cisplatin (9, 140), 5-fluorouracil (141), and cetuximab (142). Potential therapeutic targets of OSCC can also be verified in xenograft mouse models (143, 144). Researchers have also focused on invasion and metastasis (145–149), new techniques for detecting tumors (138), postoperative recurrence (127, 128), and comparisons with other models or primary tumors (132, 150). Nevertheless, xenograft mouse models are incapable of simulating the tumor microenvironment and cannot be used to study the interaction between tumors and host immunity because of the use of immunodeficient mice.

### Syngeneic OSCC Mouse Models

Syngeneic OSCC mouse models are derived from the allografts of immortalized mouse tumor cell lines, chemical carcinogen-induced spontaneous mouse tumors, or genetically engineered mouse models (GEMMs). Syngeneic mice can effectively avoid tissue rejection and GVHD caused by the transplantation of tumor cells into allogeneic mice. The models also have the inherent advantages of transplanted tumor models, such as the high speed of establishment, high consistency, and high stability. These models can better reflect the tumor microenvironment and more comprehensively simulate the complexity of the tumor. Therefore, they are widely used in the study of oncoimmunology and show great potential in the development of novel therapy for OSCC, especially immunotherapy.

#### Syngeneic Models Derived From Murine OSCC Cell Lines

The principle of syngeneic models derived from murine cell lines is similar to that of the CDX model. Various murine cell lines have been used to establish syngeneic OSCC mouse models, including murine squamous cell carcinoma SCC7 (151, 152), murine oral cancer (MOC) cell lines (153), and MOC1 (154), MOC2 (155), and murine OSCC Sq-1979 cells (156). Moroishi et al. (157) subcutaneously transplanted 1 × 10^5^ SCC7 cells into both back flanks of C3H/HeOu mice and discovered that the tumor growth was aggressive, while all of the mice transplanted with LATS1/2 dKO SCC7 cells were tumor-free. Similarly, Dong et al. (152) subcutaneously injected 1 × 10^6^ SCC7 cells into the right abdomen of C3H/HeJ mice to assess the therapeutic effect of the tumor-derived autophagosome vaccine (DRibble). To evaluate the antineoplastic effect of near-infrared photoimmunotherapy (NIR-PIT) produced by conjugating IR700DX (a photo-absorber) with anti-CD44 monoclonal antibodies, Nagaya et al. (153) built syngeneic models by subcutaneously injecting immunogenic MOC1 cells (2.0 × 10^6^), moderately immunogenic MOC2-luc cells (1.5 × 10^5^), and poorly immunogenic MOC2 mKate2 cells (1.5 × 10^5^) into C57BL/6 mice. The authors ultimately demonstrated that NIR-PIT could significantly inhibit tumor growth in the three models. Similarly, Adachi et al. (156) subcutaneously injected 1 × 10^7^ Sq-1979 cells into the posterior neck area of C3H/HeN mice to examine the genetic changes during the development of OSCC. In summary, these different murine OSCC cell lines were used to generate stable and straightforward syngeneic OSCC models successfully.

#### Syngeneic Models Derived From 4NQO-Induced Murine OSCC

Similar to the construction of the PDX model, Chen et al. (158) induced C57BL/6 mice with 4NQO (100 μg/mL in drinking water) for 16 weeks, and the mice were sacrificed at the 28th week to establish the mouse tongue squamous cell carcinoma cell lines MTCQ1 and MTCQ2. After that, the cells were injected into the flanks and the tongues of C57BL/6 mice to establish ectopic and orthotopic mouse models, respectively. Compared with the human SAS tongue SCC cell line, the proliferation of MTCQ cells was lower, but the migration and invasion abilities were much higher than those of SAS cells. A subclone of GFP-labeled MTCQ1 cells was identified by immunostaining and fluorescence imaging, and extensive cervical lymph node metastasis and lung metastasis were discovered. Some treatment methods, such as miRNAs (particularly miR-134), cisplatin treatment, and anti-PD-L1 immunotherapy, also had therapeutic effects on this model. Besides, Chen et al. (159) used 4NQO-induced OSCC transgenic mice to establish a syngeneic model. K14-EGFP-miR-211 transgenic mice were induced by 4NQO (100 μg/mL in drinking water) for 16 weeks and then sacrificed at set times. After that, the cell lines, designated MOC-L1 to MOC-L4, were established from the dissections of OSCC lesions on the dorsal tongue surface. The cell lines were used to obtain orthotopic xenografts and perform real-time *in vivo* tumor imaging by injecting 5 × 10^6^ cells into the central tongue portion of C57BL/6 mice. Additionally, the cells were used to analyze distant metastasis and assess the therapeutic efficacy of cisplatin.

The immune system is a powerful weapon against a tumor, and the emergence and development of oncoimmunology have made conquering cancer an achievable goal. Syngeneic OSCC mouse models are feasible tools for oncoimmunology, but the major issue is that the models represent oral cancer in mice and form murine tumors with murine targets. There are significant differences in the composition and reaction mechanisms between mice and humans, and some targets in humans do not exist or respond in mice.

### Humanized Mouse Models

The humanized mouse, which is developed by transplanting functional human cells or tissues into immunodeficient *Il2rg*^*null*^ mice, is an emerging preclinical model for studying human disease (160). For the study of tumors, current research focuses on [1] the establishment of novel tumor models (such as humanized CDX tumor models and humanized PDX tumor models) that are associated with human tumor-immune system interactions and [2] the exploration of therapy (including NK cell therapy, T cell editing, cytokine therapy, co-stimulatory enhancement, and checkpoint inhibitors) (161).

**Figure 5:**
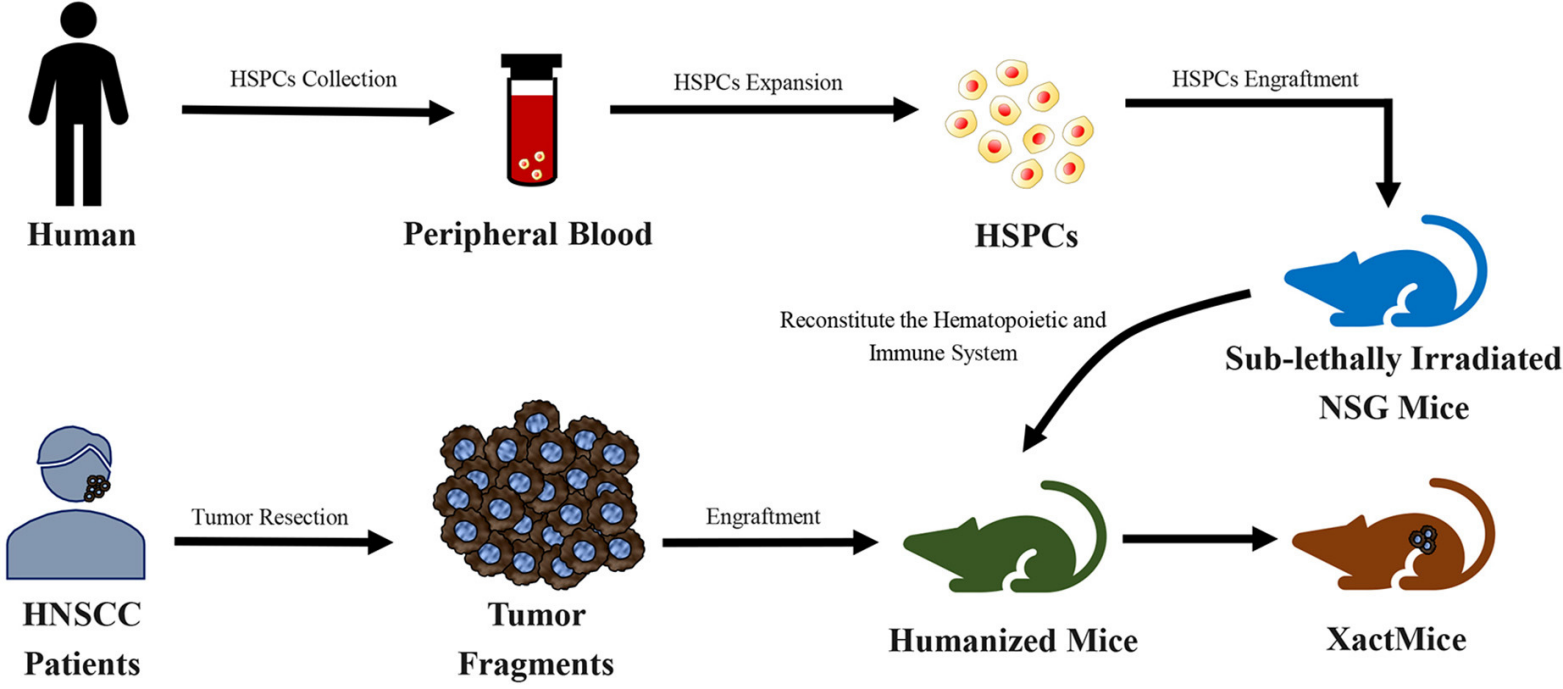
Schematic diagram of the generation of xenochimeric mice (XactMice) (162). After the cells are harvested from either cord blood or Granulocyte Colony-stimulating Factor (G-CSF) mobilized adult peripheral blood, the human hematopoietic stem and progenitor cells (HSPCs), which contain hematopoietic stem cells (HSCs), were expanded by an *ex vivo* technique and injected into sub-lethally irradiated NOD/SCID/*IL2rg*^−/−^ (NSG) mice to reconstitute the hematopoietic and immune system. Subsequently, tumor tissue from head and neck squamous cell carcinoma (HNSCC) patients were engrafted into NSG mice to generate the XactMice.

Morton et al. (162) reconstructed the tumor microenvironment in a humanized PDX mouse model of HNSCC (Figure 5). Tumors of a xenochimeric mouse (XactMice) presented human cells derived from humanized bone marrow, infiltration of human T and B cell populations, lymphangiogenesis, cytokine expression, and a dynamic microenvironment. Therefore, the XactMice system accurately recapitulates the growth of the original tumor *in vivo*. The humanized mouse model of HNSCC was optimized in another study by Morton et al. (163). After the dual infusion of human hematopoietic stem and progenitor cells (HSPCs) and mesenchymal stem cells (MSCs), the percentage of human immune cells in the bone marrow of established mice was almost twice that in mice implanted with HSPCs alone, and mature peripheral human immune cells were 9–38-fold more abundant. The dually engrafted mice also had more regulatory T cells, cytotoxic T cells, and MSCs. Thus, the dual infusion of HSPCs and MSCs resulted in a higher degree of humanization, which further increased the accuracy of the model.

As a new model, the technology of humanized mice is relatively immature, and the existing humanized immune mouse model system undeniably has many shortcomings. For instance, the immune response in the humanized mouse may be the result of tissue incompatibility (164). The transplanted human immune cells, mainly T cells, will produce GvHD and an immune attack on recipient mice and subsequently cause their death, so the operating window for conducting experiments is short (165). Nevertheless, the humanized mouse is entirely in line with the need to build a copy of the disease, so it remains a positive direction for the development of mouse models in the future.

### Transplanted Mouse Models of HPV-Related Oral Cancer

**Figure 6:**
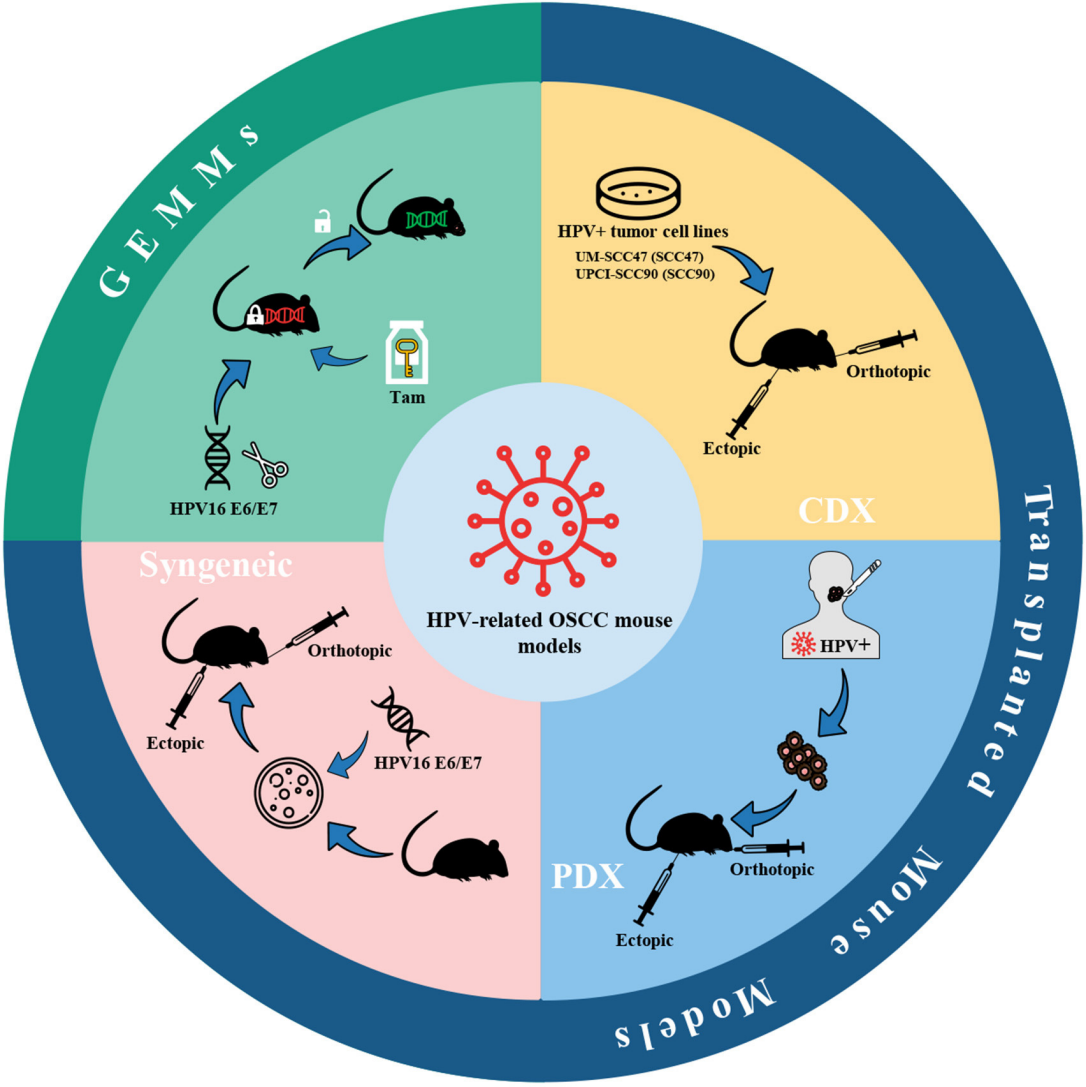
HPV-related OSCC mouse models. Mouse models of HPV-positive oral cancer can be generated by injecting HPV-positive HNSCC cell lines (CDX) or transplanting tumor fragments from HPV-positive HNSCC patients (PDX) or engrafting syngeneic HPV-positive cell lines, e.g., HPV16 E6/E7-expressing MTECs. Also, conditional GEMMs can be utilized to establish HPV-related OSCC mouse models. Adhesions: HPV, human papillomavirus; CDX, cell-derived xenograft; PDX, patient-derived xenograft; MTECs, mouse tonsil epithelial cells; GEMMs, genetically engineered models; Tam, tamoxifen.

The HPV infection is one of the risk factors for HNSCC, and the incidence of HPV-related oropharyngeal squamous cell carcinoma (OPSCC) increases sharply in men under the age of 50 (166). Within 20 years, the percentage of HPV-positive OPSCC in the United States and some European countries has risen from <20% to more than 70% (166). Given the transfer of the incidence of HNSCC to the HPV-positive population, HPV-positive oral cancer has aroused the interest of researchers, and the corresponding mouse models have been gradually developed. Currently, mouse models of HPV-positive oral cancer can be classified as transplanted models and genetically engineered mouse models (GEMMs) (Figure 6), the latter of which will be introduced in section GEMMs of HPV-related oral cancer.

The high-risk subtypes of HPV are mainly HPV16 and HPV18, while the former is considered to be associated with 90% of HPV-related cancers (167). The two proteins E6 and E7 in the early coding region of the HPV genome are highly conserved in high-risk HPV subtypes and are the most critical viral-encoded proteins involved in cancer (168). Thus, the current mouse models of HPV-related oral cancer mainly focus on the construction of cell lines or mouse strains expressing HPV16 E6/E7.

Brand et al. (169) established an HPV-related CDX model by bilaterally injecting HPV-positive HNSCC cell lines UM-SCC47 (SCC47) and UPCI-SCC90 (SCC90) into the athymic nude mice. The tumor fragments of HNSCC patients were also utilized to implant into NSG mice bilaterally to establish the PDX model. Keysar et al. (170) developed a model covering a total of 25 strains of HNSCC clinical spectrum, in which primary and recurrent tumors (including HPV-positive and HPV-negative) from HNSCC patients were implanted on mice using a modified floor-of-the-mouth (FOM) or base-of-tongue (BOT) implantation protocol to produce orthotopic tumors. Through the continuous passage of these models, the tumors maintained their original morphology, genetic characteristics, and drug susceptibilities. The gene characteristics of these tumors were following the known mutation frequencies of TP53, PI3KCA, NOTCH1, and NOTCH2. In addition, Facompre et al. (171) have also verified an elevated level of expression of p16^INK4A^ and E6/E7 virus oncogene transcripts. Meanwhile, the barriers of HPV-related HNSCC PDX models were also summarized in the research of Facompre et al. including a low engrafted rate and the formation of Epstein-Barr virus-positive (EBV+) human large B-cell lymphomas. The biological characteristics of HPV-positive HNSCC are low degree of malignancy, low invasiveness, and weak growth potential of tumor cells *in vitro*, which lead to the paucity of available HPV-positive HNSCC cell lines (172). Accordingly, selection biases against the more typical molecular characteristics of HPV-positive tumors may occur during the experiments. Likewise, using less invasive tumors as xenografts may have adverse growth characteristics and further prevent PDX from remaining stable during continuous passage *in vivo* (171).

Another HPV-related mouse oral cancer model is generated by syngeneic HPV-positive cell lines. John Lee et al. (173) transferred retroviruses expressing HPV16 E6, E7, H-Ras, and an empty control vector into mouse tonsil epithelial cells (MTECs) to construct stable murine HPV-related cell lines *in vitro*. After that, 5 × 10^5^ cells were injected into the tongue of C57BL/6 mice, or 1 × 10^6^ cells were injected into the subcutaneous tissue of the upper back near the spine. It turned out that only HPV16 E6/H-Ras MTECs and HPV16 E6/E7/H-Ras MTECs formed invasive tumors in syngeneic C57BL/6 mice at orthotopic and ectopic sites. On this basis, in order to investigate whether HPV-specific immune mechanisms can result in tumor clearance, Williams et al. (174) developed preclinical models by injecting 1 × 10^6^ HPV16 E6/E7/H-ras MTECs or HPV-negative cells subcutaneously on the right flank of C57BL/6 or SCID mice. Moreover, Mermod et al. (175) developed a novel HPV-positive HNSCC mouse model in which 1 × 10^5^ HPV16 E6/E7-expressing MTECs were transplanted into the submental region of the FOM. After that, the tumor was surgically removed to investigate the progress of postoperative primary tumor recurrence and regional lymph node metastasis. Similarly, Paolini et al. (176) established HPV16 E7 expressing mouse OSCC AT-84 cells (AT-84 E7 cells) and AT-84-E7 luminescent cells. Then 6 × 10^5^ AT-84 cells were injected into the FOM to obtain orthotopic tumors. Despite widely used, a major concern in syngeneic mouse models is the representativeness of mouse-derived tumor cells to human-derived tumor cells.

Although transplanted mouse models are currently the most commonly used animal models of HPV-related oral cancer, and their successful establishment has been documented in several publications, the lack of an appropriate HPV-positive mouse model—remaining stable during continuous passage *in vivo* and possessing more molecular characteristics with HPV-positive tumor cells of human origin—is still an obstacle to preclinical evaluation and treatment.

## Genetically Modified Mouse Models

Genetically modified mouse models, also known as genetically engineered mouse models (GEMMs), are intricate and novel animal models that have been a beneficial outcome of the development of genetic engineering technology. A review of GEMMs of OSCC was published by Ishida et al. (177), so we mainly provide an overview of GEMMs.

### An Overview of GEMMs

#### Classifications of GEMMs

GEMMs can be classified as either loss of function or gain of function. Loss of function entails gene knockout or knockdown, in which the expression of target genes is depleted or silenced. In the field of oncology, the blocked genes are typically oncogenes, tumor-suppressor genes, and metabolic genes (178). Gain-of-function studies use knock-in models of oncogene overexpression to study the function of an oncogene *in vivo*. According to the specificity, GEMMs can be classified as either conventional GEMMs or conditional GEMMs. Conventional GEMMs alter the gene of interest in every cell in the body, which is inconsistent with the reality where multiple mutational gene sites in a single cell suppress cell apoptosis and promote proliferation, resulting in tumorigenesis (179). Because of the spatiotemporal specificity of conditional GEMMs, their genes can be altered in different tissues or periods using conditional genetically modified techniques.

#### Characteristics of GEMMs

GEMMs enable the editing of specific genes through the activation or overexpression of oncogenes and the inactivation or silencing of tumor-suppressor genes. The pathological changes in these GEMMs, including hyperkeratosis, abnormal hyperplasia, carcinoma *in situ*, and invasive carcinoma (180–184), are similar to those of primary OSCC in humans and 4NQO-induced OSCC in mice. By the age of 5–6 months, histologic examination of L2D1^+^/*p53*^+/−^ and L2D1^+^/*p53*^−/−^ mice revealed hyperkeratosis, hyperplasia, severe epithelial dysplasia, and even cancer (181). Importantly, tumors of GEMMs grow in an environment with full immunity, so GEMMs are especially suitable for oncoimmunology study.

However, as a novel and promising model, there are still several barriers to the full application of GEMMs. Firstly, although the introduction of mice expressing Cre recombinase is driven by oral mucosa-specific promoters such as K5 or K14, the tumors generated by GEMMs have low specificity and appear in sites other than the oral cavity, such as the skin, tongue, esophagus, and forestomach (180, 181, 185, 186). Secondly, because of the full range of genetic changes caused by gene editing, GEMMs have a short lifespan and a high mortality rate, and highly malignant OSCC may not develop. For example, at 5 months, *p53*^−/−^ and L2D1^+^/*p53*^−/−^ mice had to be euthanized as a result of the morbidity caused by systemic lymphomas or sarcomas (181). Thirdly, the development of the human tumor is caused by mutations in a small number of cells surrounded by normal cells, while the introduction of exogenous genes or the knockout of endogenous genes in GEMMs will occur in all (conventional GEMMs) or most (conditional GEMMs) of the cells. Human tumors are usually accompanied by multiple mutations and metastases, which is not the case with GEMMs (187). Finally, the role of altered genes is questionable in the occurrence and development of OSCC. *Kras*, one of the most frequently used mutant genes in GEMMs of OSCC, has a low mutation frequency in human HNSCC, while the *ras* family is accounting for only 5% (188, 189). Almost all HPV-positive oral cancer patients are p53 wild type (190), which indicates that GEMMs of OSCC generated by p53 gene mutation are not representative of HPV-positive oral cancer.

#### Methods of GEMMs

C57BL/6 is the first mouse strain to have its complete genome sequenced and is regarded as a “standard” inbred line, which can provide a genetic background for many mutant genes. Therefore, C57BL/6 is widely used as a transgenic mouse model to mimic human genetic defects in genetic experiments. Additionally, Balb/c mice can also be used as the genetic background of GEMMs (154).

**Figure 7:**
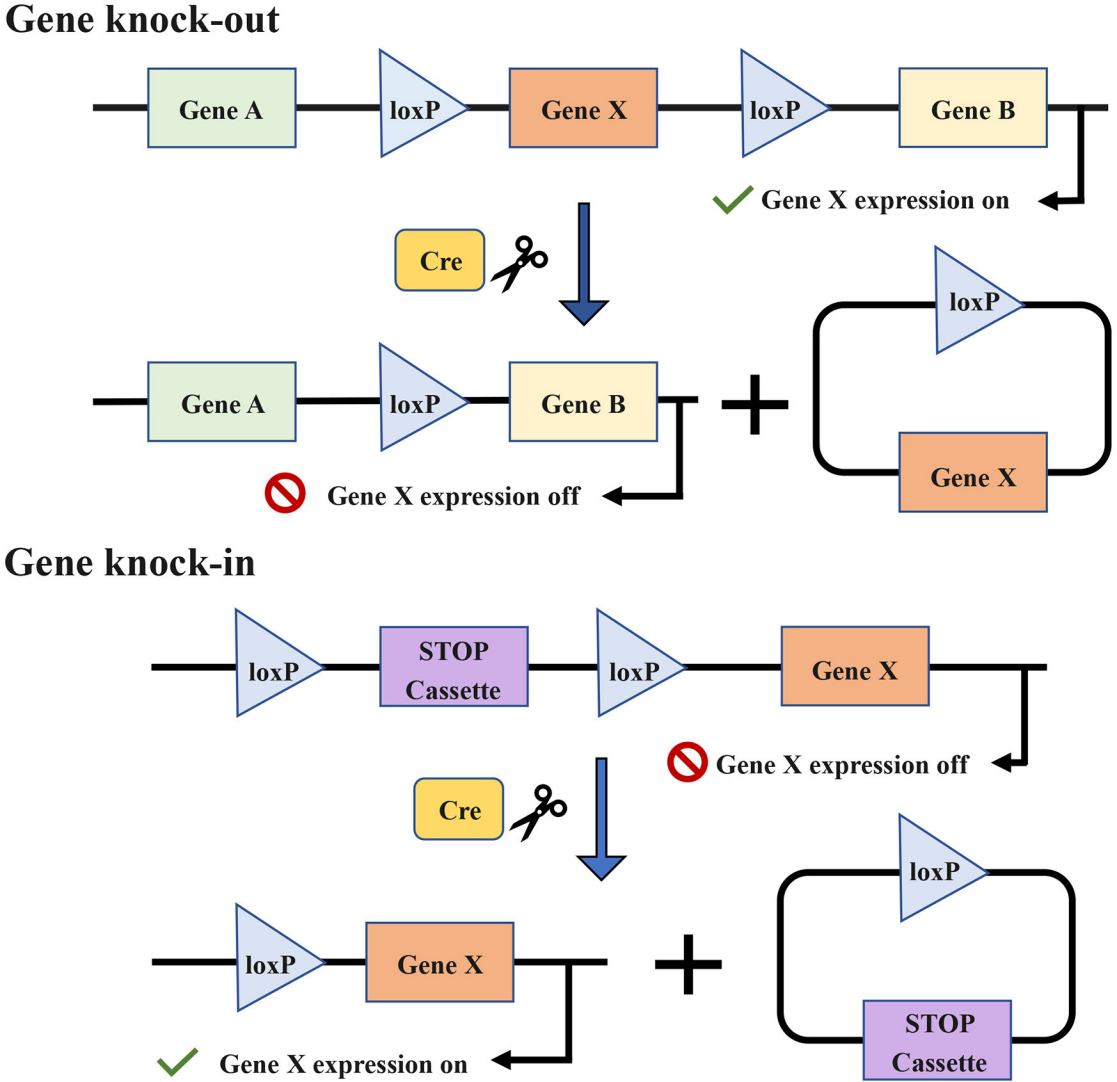
The target gene X is knocked out or knocked in via the Cre-loxP recombinase system. Cre enzymes can respectively catalyze recombination between loxP sites oriented in the same direction and flank the target gene. The introduction of Cre recombinase can cause the knock-out of the gene X. The conditional knock-in of the gene X requires another sequence named strong translational and transcriptional termination (STOP), which can terminate the expression of gene X. When Cre recombinase is present, the STOP cassette can be removed and then gene X is expressed.

The specificity of conditional GEMMs is controlled by the appearance of a recombinase, for example, the Cre-loxP recombinase system (Figure 7) (191, 192). The temporal specificity of conditional GEMMs is achieved through inducible promoters, which are regulated by exogenous chemicals, such as tamoxifen (180) and RU486 (185, 193). When the chemicals are present or removed, the promoters are activated, and then the expression of downstream target genes is changed (194, 195). The spatial specificity or tissue specificity in conditional GEMMs is determined by Cre transgenes that are expressed from tissue-specific promoters (29). For the oral cavity, the known optimal promoters are the keratin 5 (K5) and keratin 14 (K14) promoters (177). K5 is expressed within the basal layer of the tongue and forestomach stratified squamous epithelia, while K14 has been found to be expressed in the basal layer of the oral mucosa and tongue (185). Furthermore, a promoter of the Epstein–Barr virus, ED-L2, has been shown to target genes in oral and esophageal squamous epithelial cells (181, 196).

As shown in Table 5, the current GEMMs of OSCC are mainly conditional, the implementation of which mostly depends on the hybridization of the Cre mouse and gene-floxed mouse. Different genetic changes cause different cancer rates, and the carcinogenic time ranges from 10 weeks to 16 months. Generally, the activation of oncogenes or inhibition of tumor-suppressor genes accelerates the progression of carcinogenesis, while the activation of tumor-suppressor genes or the knockout of oncogenes will slow down or even halt carcinogenesis.

**Table 5:** GEMMs for OSCC.

**Mouse strains**	**Brief methods**	**Tumor formation/ conclusions**	**References**
L2D1^+^/*p53^+/−^* and L2D1^+^/*p53^−/−^* mice	The Epstein-Barr virus ED-L2 promoter (L2) was used to generate the L2-cyclin D1 (L2D1^+^) mouse, which was crossed L2D1^+^ mice with *p53^+/−^* and *p53^−/−^* mice	Both models developed invasive oral-esophageal SCC by 6 months	(181)
LSL-*Kras* ^G12D^; K5 or K14-CrePR1	The *Kras*^*G*12*D*^ oncogene driven by the K5 or K14 promoter was distributed under the control of Cre recombinase fused to a mutant of the human progesterone receptor	Administration of RU486 for 16–24 weeks induced the overexpression of oncogenic *K-ras*^*G*12*D*^ in the oral epithelium of mice and resulted in the development of squamous papillomas in the oral cavity.	(185)
*Tgfbr1/Pten* 2cKO mice	The *Tgfbr1^*flox*/*flox*^* mice and *Pten^*flox*/*flox*^* mice crossed with mice driven by *K14-CreER^*tam*^*	After tamoxifen (tam) induction for 10 weeks, the *Tgfbr1/Pten* 2cKO mice developed cancer and precancerous lesions in the oral epithelium.	(180)
*p53*^*R*172*H*^; K5-CrePR1 and *p53^*flox*/*flox*^;* K5-CrePR1 mice	The Neo-*p53*^*R*172*H*^ and *p53^*flox*/*flox*^* mice crossed with K5-CrePR1 transgenic mice	By 15–16 months, *p53*^*R*172*H*^; K5-CrePR1 (16%) and *p53^*flox*/*flox*^*; K5-CrePR1 (25%) mice developed OSCC	(182)
LSL-*Kras*; iHPV-Luc; K14-CreER^tam^ mice (KHR mice) and LSL-*Kras*; K14-CreER^tam^ mice (KR mice)	The iHPV-Luc transgenic mice were established by knocking-in HPV16 E6E7 and a luciferase reporter on an FVB background. K14-CreER^tam^ mice were backcrossed to the FVB background and then bred with iHPV-Luc mice to generate K14-CreER^tam^ × iHPV-Luc (KH) mice. After that, KH mice were bred with LSL-*Kras* mice to produce KHR or KR mice.	Tamoxifen induced oral tumors in KR and KHR mice and the bioluminescence signal of KHR mice maximized 74.8 times higher than that of untreated mice.	(197)
HPV16 E7iresE6; *PIK3CA*^*E*545*K*^; KRT14-CreER^tam^ mice	Combined Rosa26-LSL-E7iresE6 and mutant PIK3CA (Rosa26-LSL-*PIK3CA*^*E*545*K*^) under the control of KRT14-CreER^tam^ using intra-lingual tamoxifen delivery method	After the administration of tamoxifen for 6–8 weeks, oropharyngeal tumors developed with about 40% penetrance (1–2 tumors/tongue)	(198)

#### Usage of GEMMs

At present, several studies have induced oral carcinogenesis by changing the expression of oncogenes or tumor-suppressor genes and identified the links between genes and cancer. The simple GEMMs can be used to reveal novel mechanisms of carcinogenesis and will be an essential tool in the study of oral cancer-related gene mutations or proteins in the future. However, the high mortality caused by unexpected primary tumors limits its application in highly malignant OSCC.

### 4NQO-Induced Combined With GEMMs

Given that mice induced by 4NQO can develop spontaneous OSCC and precancerous lesions, some studies have combined 4NQO induction with GEMMs. This combined model integrates the advantages of both methods. Wild-type mice were used as a control in the study of this combined model. The wild-type and genetically modified mice were induced by 4NQO (50, 100, or 150 μg/mL in drinking water) for 8–30 weeks (Table 6). The effect of 4NQO on both mice reflected the effect of gene changes on OSCC, such as susceptibility and resistance.

**Table 6:** 4NQO induced combined with GEMMs for OSCC.

**4NQO application and treatment period (/weeks)**	**Mouse strains**	**Conclusions**	**Ref**.
50 μg/mL 4NQO in drinking water for 16 or 30 weeks	Ndrg2-deficient mice (*Ndrg2^+/−^* and *Ndrg2^−/−^* mice)	Tumors in Ndrg2-deficient mice were significantly developed faster and larger.	(82)
100 μg/mL 4NQO in drinking water for 8 weeks	Heterozygous p53 knockout mice (*p53^+/−^)*	Anti-PD-1 can prevent OSCC development and progression.	(89)
150 μg/mL 4NQO in drinking water for 18 weeks	*Nlrp3^−/−^* and *Caspase1^−/−^* mice	NLRP3 inflammasome promoted 5-FU resistance of OSCC *in vivo*.	(141)
100 μg/mL 4NQO in drinking water for 16 weeks	K14-EGFP-miR-211 transgenic mice tagged with GFP	MicroRNA-211 enhances the oncogenicity of carcinogen-induced oral carcinoma by repressing TCF12 and increasing antioxidant activity.	(199)
100 μg/mL 4NQO in drinking water for 16 weeks	K14-EGFP-miR-31 transgenic mice tagged with GFP	The transgenic mice had a higher susceptibility for 4NQO-induced oral and esophagus tumorigenesis.	(200)
50 or 200 μg/mL in drinking water for 28 weeks	*CCL3^−/−^* mice; *CCR5^−/−^* mice; *CCR1^−/−^* mice	SCC tumor formation is reduced in CCL3 and CCR5 deficient mice.	(201)
100 μg/mL 4NQO in drinking water for 16 weeks	*Gal3^−/−^* male mice	In *Gal3^+/+^* mice, the Hh signaling pathway might play an essential role in tongue carcinoma development.	(202)
50 or 200 μg/mL 4NQO in drinking water for 28 weeks	*ACKR2^−/−^* mice	No differences in the SCC incidence comparing wide-type and *ACKR2^−/−^* treated mice.	(203)

In the combined model, in contrast to simple 4NQO induction, specific gene deletion or overexpression can be studied; compared with simple GEMMs, more specific OSCC can be induced, and genetic changes that are not directly carcinogenic can be investigated. This model can be used to study the role of miRNA (199, 200), proteins (82, 184, 203), and pathways (201, 202) involved in the carcinogenesis of OSCC. Furthermore, the knock-in of green fluorescent protein can be used to label cells with specific gene changes (158, 199).

### GEMMs of HPV-Related Oral Cancer

By utilizing the tamoxifen-regulated Cre recombinase system, Zhong et al. (197) targeted HPV-16 oncogenes E6 and E7 and a luciferase reporter gene (iHPV-Luc) in epithelial cells of transgenic mice and established a preclinical model of autologous HPV-positive oral tumors (Table 5). Tamoxifen treatment in this model generated the development of oral tumors that detected the expression of HPV biomarkers p16 and MCM7, PIK3CA, and PTEN mutations following HNSCC sequencing data and an active mTOR-PI3K pathway. However, this GEMM still has some limitations in accurately reflecting the clinical conditions of HNSCC patients (197). First, Mutant *Kras* was required for the development of HPV-positive tumors, while the *ras* family mutations account for only about 5% of HNSCC cases. Second, the expression of HPV oncogene was artificially driven by a cytomegalovirus promoter and did not reflect human HPV positive tumors. Finally, HPV-positive mice developed oral tumors rather than oropharyngeal cancers commonly seen in HNSCC patients. Caper et al. (198) established a novel inducible GEMM of HPV-related OPSCC, in which the HPV16 oncogenes were activated in a tissue-specific and temporal manner *in vivo* (Table 5). The expression of HPV16 E6/E7 and the tissue-specific expression of mutant PIK3CA^E545K^ were induced by intra-lingual tamoxifen delivery, which replicated the histological and molecular characteristics of human HPV-positive OPSCC, including robust immune cell infiltration. Meanwhile, oropharyngeal lesions were accompanied by robust S6 phosphorylation at Ser235/236 in oropharyngeal tumors and low-level ERK1/2 activation (Thr202/Tyr204 phosphorylation).

## Conclusions

In conclusion, mouse tumor-bearing models provide a preclinical study platform and make a significant contribution to the development of a cure for OSCC. A comprehensive understanding of model establishment is crucial for thoroughly studying OSCC. While it is impossible to replicate the actual situation of OSCC absolutely, mouse models can come very close. Thus, each model should be considered on the premise of its shortcomings, and the appropriate model should be chosen according to their overall advantages. Furthermore, choosing the appropriate model based on the specific research purpose can improve research efficiency. In a single research study, several types of models can be mixed according to the research purpose to ensure the scientific robustness and correctness of the results.

## Author Contributions

QL, HD, YM, and YN: conceptualization. QL, HD, and GY: literature search. QL and HD: writing. HD: figure rendering. QL: table editing. QL, HD, GY, YS, YM, and YN: manuscript correction. All authors read and approved the final manuscript.

### Conflict of Interest

The authors declare that the research was conducted in the absence of any commercial or financial relationships that could be construed as a potential conflict of interest.
